# Predictors of Cardiovascular Events in Hypertensive Patients with High Cardiovascular Risk

**DOI:** 10.3390/medicina56040182

**Published:** 2020-04-16

**Authors:** Ivan Tasić, Svetlana Kostić, Nikola M. Stojanović, Dragan Djordjević, Dragan Bogdanović, Marina Deljanin Ilić, Milan Lović, Viktor Stoičkov, Srdjan Aleksandrić

**Affiliations:** 1 Faculty of Medicine, University of Niš, 18000 Niš, Serbia; dr.ivan.tasic@gmail.com (I.T.); ddj964@gmail.com (D.D.); marina.deljanin.ilic@medfak.ni.ac.rs (M.D.I.); viktorsss@sbb.rs (V.S.); 2 Institute for Therapy and Rehabilitation, 18000 Niška Banja, Serbia; kostic.ceki@gmail.com (S.K.); milan.lovic@gmail.com (M.L.); 3 State University of Novi Pazar, 36300 Novi Pazar, Serbia; draganbogdanovic@gmail.com; 4 Division of Cardiology, Clinical Center Serbia, 11000 Belgrade, Serbia; srdjanaleksandric@gmail.com

**Keywords:** hypertension, high cardiovascular risk, follow-up, predictors

## Abstract

*Background and objectives:* A long-term therapeutic strategy in hypertensive patients equally depends on measured arterial blood pressure values and total determined cardiovascular risk. The aim of the adequate hypertensive patient treatment is both the reduction in arterial blood pressure and the reduction of all preexisting modifiable risk factors, prevention of target organs damage, and adverse cardiovascular events. The aim of this study was to determine independent predictors of cardiovascular events in patients with hypertension and high cardiovascular (CV) risk, and whether the modifiable risk factors could affect long-term prognosis in the studied population. *Materials and Methods:* This prospective study included 142 hypertensive patients (65% females), mean age 63.1±8 years, with high CV risk. Each participant was followed for 6.2 years. *Results:* During the follow-up period, the incidence of non-fatal and fatal CV events was 19.7%, CV mortality 7%, and total mortality 9.9%. Our multivariate analysis showed that plaques in both carotid arteries (*p* = 0.042), diabetes mellitus (*p* = 0.042) and cholesterol at the beginning of the study (*p* = 0.016) were significantly associated with an increased risk of CV events. Patients’ age (*p* = 0.009), intima-media thickness (*p* = 0.001) and diabetes mellitus (*p* = 0.042) were significantly associated with an increased risk of CV mortality, and age (*p* = 0.007) and cholesterol (*p* = 0.002) were independent variables significantly associated with increased total mortality rates. *Conclusions:* The results of the present study showed that the main predictors of adverse CV events in high-risk hypertensive patients were years of age, cholesterol levels, diabetes, intima-media thickness, and carotid arteries plaques.

## 1. Introduction

High arterial blood pressure (BP) is a major and independent predictor of heart disease, stroke, kidney failure, and premature mortality and disability [1]. Arterial hypertension is usually associated with other risk factors for CV events such as dyslipidemia, smoking, diabetes mellitus (DM), age, positive family history for CV disease, obesity (especially abdominal obesity), a sedentary lifestyle, and physical and emotional stress [2].

The more detailed examination of patients with hypertension, determination of modifiable risk factors, and estimation of target organ damage (OD) in their early phase could clearly identify those with high CV risk demanding an intensive treatment approach. This kind of examination approach is suggested in the newest guidelines for hypertension, and it has led to the prevention of major CV events and deaths [3]. Due to an increase in BP, the CV morbidity and mortality are significantly increased. These facts could be explained through the significant influence of high BP on specific organs that are especially vulnerable to the changes in BP. These changes could lead to heart remodeling and hypertrophy, atherosclerotic vascular disease, kidney disease, etc.—the term target OD [4]. Today, there are well-developed sensitive noninvasive methods (tools) for the discovery of OD in their earliest phase [4]. Previous studies demonstrated that the regression of asymptomatic target OD with antihypertensive treatments led to a reduction in CV mortality and total CV events [5,6].

The primary goal of the present study was to determine the independent predictors responsible for adverse CV events in hypertensive patients with high CV risk and whether a complex antihypertensive treatment with the control of modifiable risk factors could affect long-term prognosis in this population.

## 2. Materials and Methods

### 2.1. Study Population

The present study was designed as a prospective, single-center study conducted from January 2009, which included the patients with arterial hypertension and high CV risk but without any prior major CV event. The study protocol was presented and approved by the Medical Ethical Committees of the Institute for Therapy and Rehabilitation “Niska Banja” in Serbia (decision number: 03-4307/1 from June 2015) and the Faculty of Medicine University of Nis (Nis, Serbia). Informed consent was obtained from all patients before the commencement of the study.

An initial diagnostic evaluation of a patient with hypertension involved the following: confirmation of hypertension, CV risk assessment based on BP category, CV risk factors, asymptomatic OD and presence of DM, as well as other concomitant clinical conditions. According to the 2018 European Society of Hypertension (ESH) and European Society of Cardiology (ESC) guidelines for the management of hypertension [3] high-risk hypertensive patients have ≥3 risk factors for CV diseases, metabolic syndrome, DM type 2, and/or subclinical target OD. Subclinical OD primarily involves left ventricular hypertrophy (assessed with electrocardiography or echocardiography), vascular remodeling and atherosclerosis (detected with high-resolution ultrasound scanning), and renal damage (based on the impaired renal function) [3,7]; thus, those disorders were mainly focused on.

The screening visit included an assessment of the following:

(i) Past medical history, lifestyle variables (smoking and alcohol intake), as well as family history of hypertension, CHD, or stroke.
(ii) Height, weight, body mass index (BMI) and body surface area (BSA), BP, and heart rate. Hypertension was confirmed with systolic and diastolic measurements (over 140 mmHg and 90 mmHg, respectively) and based on anamnesis, medical records, and use of antihypertensive medication.
(iii) Fasting blood samples were taken to measure blood glucose, total cholesterol, HDL, LDL, and triglycerides. The examinees with total cholesterol over 193 mg/dL or triglycerides over 151 mg/dL, or those who had been on hypolipidemic medications, were treated as hyperlipidemic individuals. The presence of DM was confirmed based on anamnestic data, medical records, increased fasting glycemia, the use of oral hypoglycemics, or insulin therapy.
(iv) Serum creatinine and glomerular filtration rate (eGFR) was calculated from the UK eCKD Guide on the Renal Association web page using the abbreviated MDRD equation: 186 × (Creat/88.4)-1.154 × (Age)-0.203 × (0.742 if female) × (1.210 if African American) [8].
(v) Echocardiography and Color Doppler Sonography (CDS) of the carotid arteries were done during the one-month screening period and before randomization.

### 2.2. Echocardiography

The echocardiographic studies were performed in the morning, with the subject in a supine left lateral decubitus position, after 30 min of rest. The measurements of left ventricular wall thickness and chamber diameter (left ventricular internal dimension) were made in diastole in accordance with the methods outlined by the American Society of Echocardiography [9]. Left ventricular mass (LV mass) was estimated by the modified cubed formula using measurements obtained in accordance with the Penn convention [10]: LV mass (g//m^2^) = 1.04 [(left ventricular internal dimension + ventricular septal thickness + posterior left ventricular wall thickness)^3^-(left ventricular internal dimension)^3^]-13.6.

### 2.3. Color Doppler Sonography of Carotid Arteries

Color Doppler sonography (CDS) of the carotid arteries was performed using Esaote BiomedicaMy Lab60 Xvision, with a 4–13 MHz multi-frequency linear probe, and the carotid intima-media thickness (IMT) and plaques were measured as described previously [11,12]. Values of IMT >0.9 mm, the presence of plaque (if IMT was >1.5 mm), or a focal thickness increase of 0.5 mm or 50% of the surrounding IMT value, were considered pathological [11,12].

### 2.4. Follow-Up Period

After a one-month screening period, hypertensive patients with high CV risk were included in the treatment program, involving education about lifestyle changes and antihypertensive medication. The target BP value was 140/90 mmHg in patients without DM, and 140/85 mmHg in those with DM. Outpatient visits were scheduled in 1, 3, 6, 9, and 12 months, and after the last visit for every six months. Mean follow-up was 6.2 years. Echocardiography and CDS of carotid arteries were performed every 12 months.

The incidence of the following events was monitored throughout the study: (1) major cardiovascular events (including fatal and nonfatal myocardial infarction and stroke, coronary artery revascularization (percutaneous coronary intervention or coronary artery bypass graft) and cardiovascular death); (2) cardiovascular death; and (3) all-cause mortality (death resulting from any cause).

### 2.5. Statistical Analysis

All data were entered into a specifically created database and then processed in the statistical program IBM Statistical Package for the Social Sciences version 18.0 (SPSS Inc., Chicago, IL, USA). Continuous variables are expressed as mean ± SD, and categorical variables are reported as a count with percentages. Categorical variables were compared using a Chi-squared or Fischer’s exact test, depending on the group size. Normal distribution of continuous variables was confirmed by the Kolmogorov–Smirnov test. All tests were considered two-sided. The effects of the examined characteristics on study outcomes were estimated using the univariate and multivariate Cox proportional hazards model. The Backward Conditional method was used to select the independent variables that were significantly associated with the dependent variable in multivariate analysis. A two-sided *p* < 0.05 was considered to be statistically significant.

## 3. Results

### Patient Characteristics

One hundred and forty-two patients (93 females (65%), 49 males (35%), mean age 63 ± 8 years, range 43–80) with hypertension and high CV risk were included in this study. Half of the patients had grade 1 hypertension, 37% had grade 2 hypertension, and 14% had grade 3 hypertension. The BMI in female patients indicated an average overweight status, with a relatively higher prevalence of lipid disorders and DM, and a relatively low prevalence of active smoking (11.3%). About 50% of patients had formed plaques in the carotid arteries, and most of them had mild ventricular hypertrophy.

Clinical and biochemical characteristics and echocardiographic and CDS of carotid arteries parameters (LV mass index and carotid IMT) at the beginning and the end of the study are shown in Table 1. Systolic and diastolic BP, as well as the levels of LDL and total cholesterol at the final follow-up, were significantly decreased when compared to the initial screening. However, their BP and cholesterol average values at the end of the follow-up period were still above the target values. There were no significant differences in LV mass index, carotid IMT, and eGFR at the initial screening and the end of follow-up, suggesting a satisfactory level of target organ protection during the study.

A majority of the studied patients were using combined antihypertensive therapy—66.9% used three or four medications, while in some cases, patients used one (11; 7.7%) or seven drugs (5; 4.9%), respectively (Table 2). Among those on combined therapy, the highest number of patients was using angiotensin-converting enzyme (ACE) inhibitors and angiotensin receptor blockers (ARB) (85%), while the lowest percentage were prescribed calcium-channel blockers (CCB) (64.79%) (Table 2). Fixed two-drug combinations were taken by 36.6% of the studied patients.

Statins were used by 69 patients (48.6%), while non-statin anti-lipid therapy was used by 10 patients (7%) (Table 2). At the beginning of our study, 42.2% of examinees had total cholesterol above 240 mg/dL, while 37.4% had value in the range between 193 mg/dL and 240 mg/dL, and 65.5% had LDL cholesterol 100 mg/dL (Table 1). At the end of the study, 31.7% had the target total cholesterol value below 193 mg/dL, while the target LDL cholesterol value for high-risk patients of <100 mg/dL was reached by 42% of our examinees. Thirty patients (21.1%) had DM, and 22 of them used oral antidiabetic drugs (OAD), while the rest of them were on insulin alone or in combination with OAD (Table 2).

During the follow-up period (mean value 6.2 ± 1.8 years), there were 28 (19.7%) patients with major CV events, while the CV was the cause of death in 7% and the total mortality rate in this period was 9.9%. Associations of the examined clinical, biochemical and echocardiographic parameters with the major CV events were analyzed (Table 3). LV mass index, carotid IMT, plaques in the carotid arteries, diabetes mellitus, cholesterol levels, systolic BP and a decrease in eGFR (worsening of the kidney function) at the beginning of the follow-up period were found to be associated with an increased risk of major CV events (Table 3). On the other hand, female gender was associated with a decreased risk for CV events. In a multivariate analysis, the presence of plaques at two or more sites, DM, and cholesterol levels were found to be independent variables (risk factors) that are significantly associated with an increased risk for CV events, while female sex was found to be associated with a decreased risk for CV events (Table 3). Data given in Table 4 presents the results of multivariate regression analysis, which indicates that age, IMT, and DM are independent variables significantly associated with an increased risk of CV mortality.

Age, IMT, the presence of plaques either in one or both carotid arteries, DM serum cholesterol levels, and a decrease in eGFR were associated with an increased risk of all-cause mortality during the studied time period (Table 5). Additionally, multivariate analysis revealed that age and cholesterol levels were independent variables significantly associated with an increased risk of all-cause mortality (Table 5).

## 4. Discussion

After the 6.2 ± 1.8 years follow-up and statistical analysis of the data, there were 19.7% patients with a major CV event and the CV mortality was 7%, while the all-cause mortality was found to be 9.9%. Total (all-cause) mortality of 9.9% in our hypertensive patients with high CV risk is in good correlation with the range of high risk calculated SCORE [13]. Previous studies have also revealed that the years of age are an important predictive factor for CV mortality and total mortality, as well as for the CV events occurrence [14]. The average age of our patients was 63 ± 8 years, making the studied population near the age (65 years) in which most individuals are already at a (very) high CV risk.

Our study showed that the baseline (initial values) of total cholesterol levels could be regarded as important predictive parameters for CV events and total mortality. The Framingham study revealed that 31%–35% of hypertensive men and women below 65 years of age had cholesterol values exceeding 6.2 mmol/l, while the corresponding percentage of those over 65 years of age was 25%–52% [15]. In our study, we found that total and LDL cholesterol values were reduced by 15% and 13%, respectively (Table 1). Results of a large ASCOT-BPLA (Prevention of cardiovascular events with an antihypertensive regimen of amlodipin adding perindopril as required versus atenolol adding bendoflumethiazide as required, in the Anglo-Scandinavian Cardiac Outcomes Trial-Blood Pressure Lowering Arm) study claim that a 10 mg dose atorvastatin reduces the levels of total and LDL cholesterol by 24% and 35%, respectively [16]. Also, the ALLHAT (Antihypertensive and Lipid-Lowering Treatment to Prevent Heart Attack Trial) study reported total cholesterol reduction by 17.2% with pravastatin, vs. 7.6% found with usual diet after four years of study [17]. We did not perform a detailed analysis of the connection between statin use and reduction in total and LDL cholesterol levels, so we therefore cannot claim that the unexpectedly low decrease in lipid parameters is fully associated with the statin therapy.

The adherence to pravastatin treatment was found to decrease during some studies, where it dropped from 87.2% in the second year, to 80% in the fourth, and 77% in the sixth year [17]. Statins (different drugs) were administered to all patients enrolled in this study who had LDL cholesterol ≥116 mg/dL and total cholesterol levels ≥193 mg/dL at the first control, following the implementation of the dietary regime (Table 2). Statin therapy had to be withdrawn in one of the patients due to a significant increase in creatine kinase activity. From the present results, we can see that target cholesterol levels are still difficult to reach in clinical practice and that the adherence to statin therapy as a primary prevention for hypertensive patients can still be considered a problem.

Findings of this study also support previous statements that DM can be significantly associated with CV morbidity and mortality [18]. The percentage of patients with DM was 21.1% at the onset of the study and 26.8% at the end (developed in additional 8 patients). Although DM was found to be significantly associated with CV events and mortality, the same association was not found for glycemia (Table 3 and Table 4). Interestingly, the p value for glycemia in the univariant analysis was almost at the level of significance in the case when the association was estimated for CV mortality (Table 4). All patients with DM were treated with either OAD and/or insulin, which could be related to controlled glycemia. This could also explain, at least in part, the absence of association between glucose levels and CV events or mortality, while the association was significant with the dicotomic variable DM. The presence of DM and hypertension can reflect the occurrence or rapid progression of the target OD (heart, blood vessels, and kidneys), which further increases the risk of different CV events [19]. The reduction and good control of BP are especially important in hypertensive patients with DM. The latest meta-analyses have shown that antihypertensive treatment reduces the risk of mortality and cardiovascular morbidity in people with DM and a systolic BP over 140 mmHg [18]. According to the latest European guidelines for hypertension and diabetes, target BP values in patients with DM aging 18 to 65 are 120–130/70–79 mmHg, whilst in patients with DM and aging above 65, the target values are 130–139/70–79 mmHg [3,20]. In everyday clinical practice, in order to achieve target BP values, patients have to take two or more medications or combination therapy, followed by a rigorous non-pharmacological treatment regime [21]. Renin-angiotensin system blockers should be an integral part of the treatment in such patients because of their cardiovascular and nephroprotective effects [21]. Such BP value, together with these groups of drugs (renin-angiotensin blockers and other antihypertensives) were incorporated as standard in our study protocol [3,20]. More than 80% of our patients were taking renin-angiotensin blockers (Table 2), while around 67% of them were using three or more antihypertensive drugs. Baseline LVH and carotid artery disease are significant predictors of new-onset DM in a large population of treated hypertensive patients, independently of the baseline metabolic profile and therapy [19].

Atherosclerotic changes in the carotid arteries (presence of plaques) and IMT were shown to be important predictors of CV events and CV mortality in our study. Followed patients had a high prevalence of plaques in the carotid arteries—52% (i.e., an IMT of >1.5 mm) compared to the Verapamil in Hypertension and Atherosclerosis Study (VHAS) [22], which used the same criterion for IMT and found that 40% of patients had a plaque in at least one of the carotids, while only 33% had normal carotid arteries. The ELSA study reported that 82% of hypertensive patients had plaques, but here the intima-media thickness criterion of >1.3 mm was used [23]. The RIS (Risk Intervention Study) has shown that one-third of the studied patients with essential hypertension and high CV risk also had a moderate to large plaque in the carotid artery region [24]. In a meta-analysis conducted by Lorenzo et al. it is shown that IMT is significantly associated with future CV events and that an absolute difference in IMT of 0.1 mm increases the risk for future myocardial infarction by 10–15% and the risk for brain stroke by 13–18% [25].

The results of one of our earlier studies showed that the number of coronary blood vessels affected by atherosclerosis is increasing significantly with an increase in IMT by 0.729 mm [12]. Studied hypertensive patients with high CV risk had only slightly lower IMT (0.94 ± 0.25) than the coronary patients from our earlier study (0.98 ± 0.21) [12], suggesting that more intense medicamentous therapy can contribute very little to the IMT. The results of the PHYLLIS study reported that in hypertensive and hypercholesterolemic patients the administration of pravastatin alone prevents the progression of carotid IMT, similar to that seen in patients treated with hydrochlorothiazide. However, the combination of pravastatin and the ACE-inhibitor fosinopril had no additive effect on the IMT [26]. Our earlier results have demonstrated that, in hypertensive patients with LVH after nine months, the thickness of the intimomedial complex in patients using fosinopril dropped by 0.0278 ± 0.03 mm, while in the group of patients that did not use this ACE-inhibitor, its value increased by 0.078 ± 0.13 mm [27]. In spite of the firm evidence of the association of carotid artery changes (IMT and plaques) with the risk of CV events obtained in the present study and from the numerous others, we still cannot definitively conclude that a medicamentous treatment is able to produce IMT regression and thus to improve patients prognosis/survival.

Left ventricular hypertrophy (LVH) in hypertensive patients is regarded as a major risk factor for CV events. Direct LV mass measurement using echocardiography (M-mode, under two-dimensional control) is found to be a significant predictor of risk for CV morbidity and mortality [28]. Although significant correlations regarding LVH and CV complications have been previously reported [29], we were unable to find a similar correlation in the present study. This could perhaps be explained by the fact that most of the patients enrolled in the present study had mild to moderate LVH expressed as the LV mass index (Table 1). The average value of LV mass index at the end of the study was negligibly higher from the baseline value, which could mean that the instituted antihypertensive therapy prevented any further significant LVH progression. This could also be explained by the low number of patients with congestive heart failure at the end of the study (only one patient). Our preliminary reports showed that a reduction in the LV mass index was achieved by 55.9% of patients and a reduction or non-progression of IMT was achieved by only 31% [30]. Also, the regression of LVH and IMT depends on adequate and continued BP regulation, as well as on the presence and regulation of DM [26].

## 5. Conclusions

In summary, the results of our study showed that the main predictors of adverse cardiovascular events in high-risk hypertensive patients were years of age, cholesterol, diabetes mellitus, intima-media thickness, and plaques in the carotid arteries. Although the complex antihypertensive treatment and management of other risk factors were accomplished abiding by the current guidelines, the target values of cholesterol and adherence to statin therapy were the most difficult to achieve.

## Figures and Tables

**Table 1:** Clinical and biochemical characteristics of the study population at the initial screening and final follow-up.

Variable	Initial Screening	Final Follow-Up	*p*
Systolic BP (mmHg)	157.6 ± 16.6	146.1± 18.4	<0.001
Diastolic BP (mmHg)	91.2 ± 10.9	86.1± 9.3	<0.001
BMI (kg/m^2)^	28.8 ± 3.7	28.8± 4.2	>0.05
Glycemia, (mg/dL)	106.6 ± 25.9	113. ± 43.9	>0.05
Total cholesterol (mg/dL)	231.2 ± 39.4	211.5 ± 39.8	<0.001
LDL cholesterol (mmol/dL)	147.3 ± 34	128.8 ± 33.6	<0.001
HDL cholesterol (mmol/dL)	46.8 ± 11.2	49.1 ± 11.6	>0.05
Triglycerides (mmol/dL)	185.1 ± 139.1	164.8 ± 93	>0.05
Diabetes mellitus (%)	21.1	26.8	>0.05
eGFRmL/min per 1.73 m^2^	69.3 ± 17.4	70.3 ± 19.9	>0.05
LV mass index (g/m^2^)	138.9 ± 30	142.6 ± 35.5	>0.05
CIMT (mm)	0.94 ± 0.25	0.96 ± 0.24	>0.05
Plaque (%)	52.1	56.3	>0.05

The data are expressed as either means ± SD or percentages. BP—blood pressure; BMI—body mass index; LDL – low density lipoprotein; HDL – high density lipoprotein; eGFR—estimated glomerular filtration rate; LV—left ventricle; CIMT—carotid intima-media thickness.

**Table 2:** Use of antihypertensive and other types of medications.

Type of Medications	N (%)
ACEIs/ARBs	84.51
BBs	75.35
CCBs	64.79
Diuretic agents	71.83
Statins	50
Fibrates	7
Diabetes therapy	15.5
Acetylsalicylic acid	35.9

Values are expressed as percentages. ACEIs/ARBs—angiotensin-converting enzyme inhibitors and angiotensin receptor blockers; BBs—beta-blockers; CCBs—calcium-channel blockers.

**Table 3:** Association between the examined parameters and major cardiovascular (CV) events.

Parameter	HR	95.0% CI	*p*
*Univariant Cox regression analysis*			
Female gender	0.476	(0.227–0.999)	0.05
Age (years)	1.020	(0.974–1.068)	0.396
Smoking habits	1.453	(0.504–4.191)	0.489
BMI (kg/m^2^)	0.971	(0.879–1.074)	0.569
Systolic BP (mmHg)	1.033	(1.008–1.059)	0.008
Diastolic BP (mmHg)	1.008	(0.960–1.058)	0.755
Diabetes mellitus	2.225	(1.026–4.821)	0.043
Glycemia (mg/dL)	1.205	(0.959–1.514)	0.110
Total cholesterol (mg/dL)	1.592	(1.109–2.287)	0.012
LDL cholesterol (mg/dL)	1.146	(0.711–1.847)	0.577
HDL cholesterol (mg/dL)	0.932	(0.256–3.391)	0.915
Triglycerides (mg/dL)	1.079	(0.880–1.324)	0.463
eGFR (mL/min per 1.73 m^2^)	0.989	(0.967–1.010)	0.295
LV mass index (g/m^2^)	1.015	(1.004–1.026)	0.010
Intima-media thickness (mm)	10.522	(3.273–33.823)	0.000
Plaque	3.785	(1.534–9.340)	0.004
Plaque in both carotid arteries	5.263	(2.314–11.97)	<0.001
*Multivariate Cox regression analysis*			
Female gender	0.233	(0.083–0.653)	0.006
Diabetes mellitus	3.163	(1.04–9.613)	0.042
Total cholesterol (mg/dL)	1.879	(1.12–3.142)	0.016
Plaques in both carotid arteries	2.989	(1.08–8.607)	0.042

HR—hazard ratio; BP—blood pressure; BMI—body mass index; eGFR—estimated glomerular filtration rate; LV—left ventricle.

**Table 4:** Association between the examined parameters and CV mortality—univariate and multivariate analysis.

Parameter	HR	95.0% CI	*p*
*Univariant Cox regression analysis*			
Female gender	1.906	(0.404–8.985)	0.415
Age (years)	1.211	(1.078–1.360)	0.001
Smoking habits	0.945	(0.120–7.464)	0.957
BMI (kg/m^2^)	0.990	(0.841–1.167)	0.907
Systolic BP (mmHg)	1.049	(1.009–1.090)	0.015
Diastolic BP (mmHg)	0.924	(0.850–1.003)	0.060
Diabetes mellitus	4.039	(1.168–13.967)	0.027
Glycemia (mg/dL)	1.371	(0.990–1.898)	0.057
Total cholesterol (mg/dL)	1.449	(0.791–2.654)	0.229
LDL cholesterol (mg/dL)	0.820	(0.342–1.965)	0.656
HDL cholesterol (mg/dL)	1.375	(0.164–11.538)	0.769
Triglycerides (mg/dL)	0.974	(0.615–1.542)	0.909
eGFR (mL/min per 1.73 m^2^)	0.951	(0.916–0.987)	0.008
LV mass index (g/m^2^)	0.991	(0.970–1.013)	0.442
Intima-media thickness (mm)	32.810	(5.607–192.002)	0.000
Plaque in both carotid arteries	5.019	(1.292–19.499)	0.020
Plaque	4.165	(0.883–19.647)	0.071
*Multivariate Cox regression analysis*			
Age (years)	1.155	(1.037–1.287)	0.009
Intima-media thickness (mm)	41.211	(4.614–368.126)	0.001
Diabetes mellitus	4.123	(1.003–1.160)	0.040

HR—hazard ratio; BP—blood pressure; BMI—body mass index; eGFR—estimated glomerular filtration rate; LV—left ventricle.

**Table 5:** Association of the examined parameters with all-cause mortality: results of the univariate and multivariant Cox regression analysis.

Parameter	HR	95.0% CI	*p*
*Univariant Cox regression analysis*			
Female gender	0.844	(0.227–0.999)	0.761
Age (years)	1.140	(1.048–1.239)	0.002
Smoking	1.435	(0.321–6.416)	0.636
BMI (kg/m^2^)	0.983	(0.855–1.131)	0.813
Systolic BP (mmHg)	1.022	(0.988–1.058)	0.212
Diastolic BP (mmHg)	0.938	(0.798–1.003)	0.062
Diabetes mellitus	3.075	(1.066–8.870)	0.038
Glycemia (mg/dL)	1.271	(0.947–1.707)	0.111
Total cholesterol (mg/dL)	1.820	(1.098–3.017)	0.020
LDL cholesterol (mg/dL)	1.146	(0.576–2.282)	0.698
HDL cholesterol (mg/dL)	0.574	(0.087–3.777)	0.564
Triglycerides (mg/dL)	1.066	(0.787–1.444)	0.679
eGFRmL/min per 1.73 m^2^	0.964	(0.934–0.994)	0.021
LV mass index (g/m^2^)	1.007	(0.991–1.024)	0.396
Intima-media thickness (mm)	8.147	(1.473–45.042)	0.016
Plaque	3.908	(1.089–14.029)	0.037
Plaques in both carotid arteries	5.510	(1.722–17.632)	0.004
*Multivariate Cox regression analysis*			
Age	1.117	(1.031–1.211)	0.007
Total cholesterol (mg/dL)	2.526	(1.398–4.566)	0.002

HR—hazard ratio; BMI—body mass index; BP—blood pressure; eGFR—estimated glomerular filtration rate; LV—left ventricle.
